# A study on refined curing control of precast segmental concrete for prefabricated railway bridges

**DOI:** 10.1038/s41598-026-41606-z

**Published:** 2026-04-29

**Authors:** Rong He, Kangkang Zhang, Wei He

**Affiliations:** School of Civil Engineering and Transportation, North China University of Water Resources and Electric Power, Zhengzhou, 450045 Henan PR China

**Keywords:** Railway bridge, Concrete, Hydration heat, Influencing factor, Refined curing, Engineering, Materials science

## Abstract

To ensure the prefabrication quality of concrete segmental beams for assembled railway bridges, this study investigates the main factors affecting the casting and curing of such beams. Using the Zheng-Xu regional railway bridge as a case study, this study analyzes the impact of environmental temperature, formwork materials, formwork thickness, pouring temperature, wind speed, and prestressed ducts on the thermal-structural coupled stresses in the beams, based on meteorological conditions at the prefabrication yard. A detailed concrete curing control plan is proposed. The research results show that formwork materials and pouring temperature have a significantly greater influence on the early thermal effects of prefabricated concrete segmental beams than other factors. Formwork materials exhibit the highest sensitivity to temperature and temperature stress, with sensitivities of 17.8% and 36.1%, respectively, followed by pouring temperature, which has sensitivities of 12.7% and 21.7% to temperature and temperature stress, respectively, and is positively correlated with peak temperature and peak stress. Wind speed and prestressed ducts are sensitive to temperature stress, with their sensitivities being less than 5%. Prestressed ducts promote internal heat dissipation in concrete segmental beams, reducing the internal peak temperature and the temperature difference between the interior and exterior, thereby lowering the risk of cracking. The better the thermal insulation performance of the formwork materials, the later and higher the temperature peak. Additionally, the thickness of the formwork materials has a greater impact on the temperature and stress of the segmental beam concrete. Based on the identified temperature field and thermal stress patterns for each significant factor, this study proposes detailed curing plans, including winter steam curing and summer spray curing. These strategies effectively reduce the internal-external temperature difference, minimize surface stresses, and mitigate the risk of cracking. The quality of prefabricated concrete segmental beams confirms the scientificity and rationality of the fine-curing control plan, which can serve as a reference for the detailed control of prefabrication and curing.

## Introduction

Segmental precast and assembled railway bridges are commonly constructed using large-scale concrete box sections. Given that concrete has relatively low thermal conductivity, the early-age temperature field after casting is strongly influenced by the curing ambient temperature, formwork materials, formwork thickness, casting temperature, wind speed, and prestressed ducts. In the initial casting stage, concrete has a low elastic modulus and insufficient strength. When a significant temperature gradient develops between the interior and exterior of the box girders, excessive thermal stresses may arise, leading to early-age cracking and thereby compromising the structural safety and durability of the bridges^1,2^. Therefore, investigating the effects of these factors on the early-age temperature and stress fields of segmental precast box girders during curing is essential. Such research provides the basis for developing fine curing control measures for segmental precast concrete beams, which are critical for ensuring fabrication quality, preventing cracking, and enhancing the long-term performance of prefabricated bridges.

Regarding the early-age temperature fields of concrete box girders, numerous scholars have conducted relevant research. Lu Wenliang and He Guofeng^3,4^ investigated the evolution of early-age temperature fields in concrete box girders experimentally. Chen Zhiqing and Zhang Guoyun^5,6^ conducted numerical analyses of hydration heat temperature fields using finite element software. Li Fulu^7^ examined the effects of material properties, structural parameters, and environmental factors on the hydration heat of concrete box culverts in cold regions. Hui Yingxin^8^ studied the mechanisms by which ambient and casting temperatures influence the stress state of web plates in box girders. Jia Weizhong et al.^9^ explored the control of thermal cracking induced by hydration heat, focusing on casting temperature, curing methods, and initial prestressing levels. Li Xiangdong et al.^10^ analyzed the effects of thermal conductivity, hydration parameters, elastic modulus, creep, and foundation restraint on temperature rise. Liang Dong^11^, Zeng Y^12,13^, Tong Zhi^14^, M. Gi^15^, and Y. Cai^16^ investigated the effects of material properties and ambient temperature on hydration heat temperature fields. Chen Xiong et al.^17^ studied the effects of slenderness ratio, heat release rate, and casting temperature on arch deformation in segmental girders. Nassif^18^ investigated the effects of low-temperature curing on the stiffness and strength of concrete, while Lee^19^ examined the compressive strength development of concrete under different curing temperatures. Overall, existing research has primarily focused on the early-age temperature fields of monolithically cast box girders or the effects of environmental temperature variations and intrinsic material properties. However, relatively few studies have systematically investigated the thermo-mechanical behavior of segmentally precast box girders under different curing conditions, particularly considering the coupled effects of formwork materials, formwork thickness, casting temperature, wind speed, and prestressing ducts on hydration heat and temperature fields.

This study focuses on the segmental precast box girders of the Zhengzhou–Xuchang intercity railway bridge. It investigates the effects of curing conditions, formwork materials, formwork thickness, casting temperature, wind speed, and prestressing ducts on the temperature–stress fields of segmental concrete beams after casting. Based on these analyses, this study proposes fine curing control measures for precast segmental concrete beams.

## Project overview

**Fig. 1:**
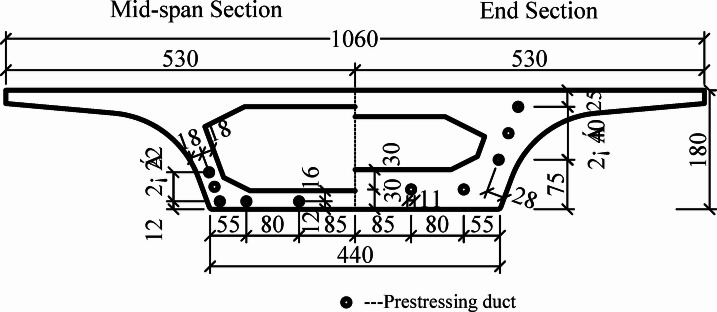
End and mid-span cross-sections of segmental beam (Unit: cm).

The Xuchang section of the Zhengzhou–Xuchang intercity railway extends from the municipal border of Zhengzhou and Xuchang in the north to Xuchang East Station in the south, with a total length of 33.78 km. The section comprises 27.823 km of elevated line, 1.941 km of surface line, and 4.016 km of underground section. The elevated portion primarily adopts simply supported, double-track, segmental, precast, prestressed concrete box girders. Each girder has a width of 10.6 m, a bottom slab width of 4.4 m, and a height of 1.8 m. The cross-section is a single-cell box section, with top-and-bottom slab thicknesses of 25 cm and 28 cm, respectively, and a web thickness of 36 cm at midspan. The beam sections at the beam ends and midspan are shown in Fig. 1. At the girder ends, the top slab, bottom slab, and web are thickened to 40 cm, 60 cm, and 60 cm, respectively. Prestressed ducts are arranged along the bottom slab, web, and their intersections. The prestressed ducts have a diameter of 11 cm.

The segmental girders are classified into three types: standard, transition, and end ones. The standard and transition segments of the girders are each 2.5 m long, while the end segments are 2.45 m long. A 30 m segmental girder consists of 12 segments, including 2 end segments, 2 transition segments, and 8 standard segments. A 25 m segmental girder comprises 10 segments: 2 end segments, 2 transition segments, and 6 standard segments. Along the entire line, 736 box girders will be erected, including 640 of 30 m span and 96 of 25 m span, resulting in a total of 8,640 precast segments. All segments are prefabricated at the Xuchang casting yard using C50 concrete and HPB300/HRB400 steel reinforcement. Given the large number of girders, the extended casting period of over one year, and the significant variations in ambient conditions during construction, a refined curing control scheme for segmental concrete must be developed based on local meteorological data. This scheme aims to prevent early-age cracking and ensure the quality and durability of the precast girders.

### Local meteorological conditions

**Fig. 2:**
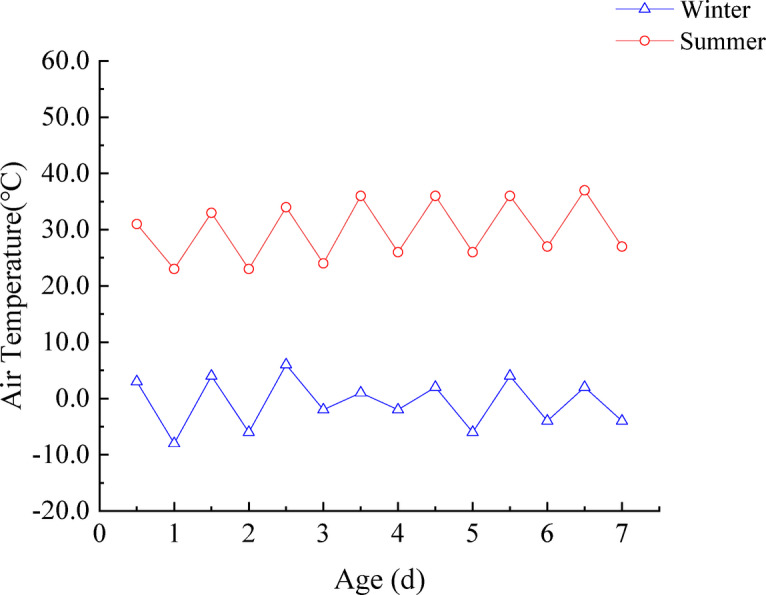
Air temperature at the precast yard (Unit: ℃).

The project is located in Xuchang City. According to meteorological data collected over the past 50 years by the local weather bureau, the lowest temperatures in Xuchang typically occur in January, while the highest temperatures are recorded in July. To represent realistic environmental conditions for concrete casting, the minimum and maximum air temperatures were selected based on the lowest and highest daily values observed over a continuous seven-day period in January and July, respectively, during the three years preceding construction. These data were used to define the representative winter and summer temperature conditions at the precast yard, as illustrated in Fig. 2.

### Preliminary curing scheme

To prevent segmental beams from cracking during prefabrication and to ensure construction quality, a preliminary concrete curing scheme was developed based on local meteorological conditions at the precast yard and on practices from similar engineering projects^20^. The details of the proposed curing methods are as follows:

During the summer, a water-spray curing method is adopted. Immediately after concrete casting, high-pressure water jets are used to spray the girder surface, cooling it and retaining moisture. The formwork is removed after 3 days, with continuous mist spraying both before and after removal to reduce surface temperatures. The total curing duration is no less than 30 days. During winter, steam curing is applied due to the low ambient temperature in Xuchang. A thermally insulated enclosure made of fire-resistant rock wool and colored steel panels is used to maintain the temperature during steam heating. Steam curing begins approximately four hours after casting. Formwork removal is carried out after three days, with additional thermal insulation and steam heating applied before and after demolding to prevent thermal shock and ensure adequate strength development.

## Model development

### Finite element model

**Fig. 3:**
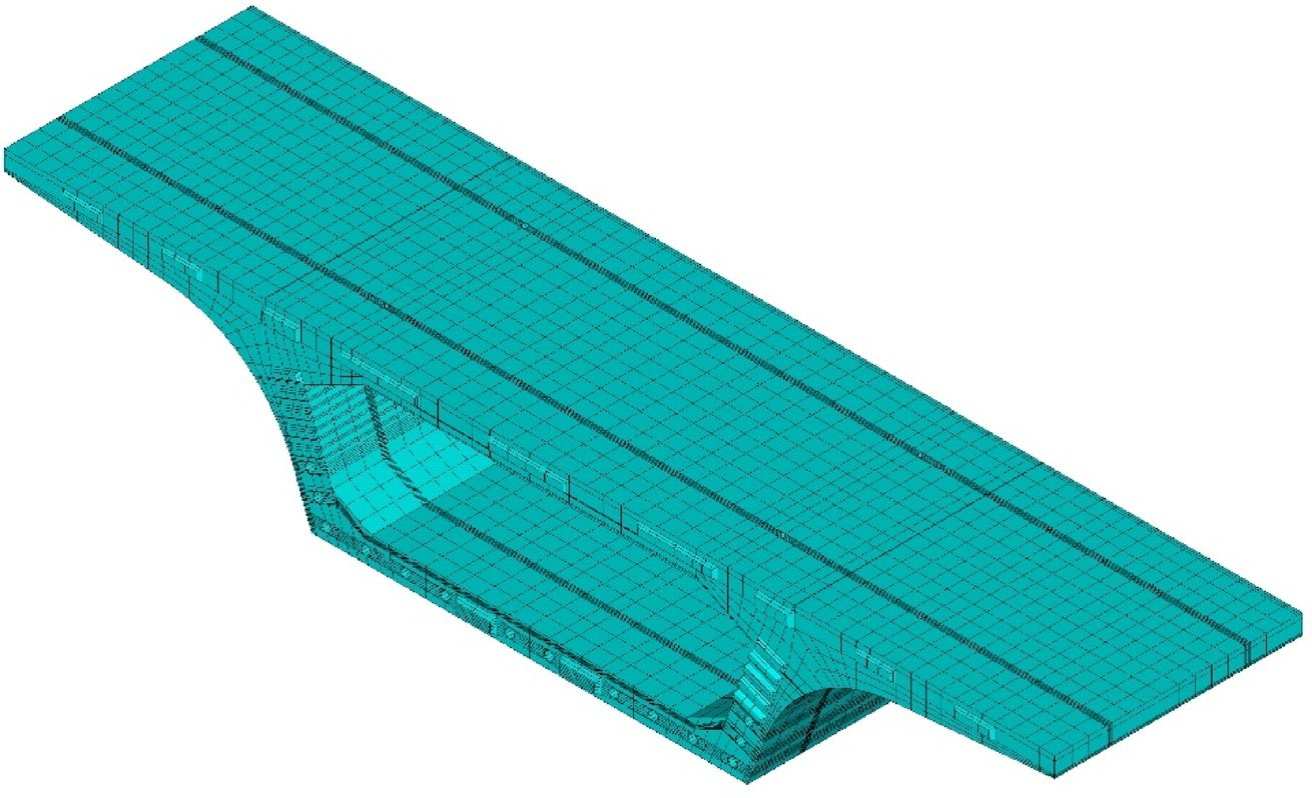
Finite element model of the midspan segment.

A finite element (FE) model of a segmental box girder is developed using ANSYS software from the detailed design drawings. The physical parameters of each constituent material are determined from measured experimental or specified values. The segmental girder segments are simulated using 3D solid elements. The SOLID70 element is employed for the corresponding temperature field analysis, while the SOLID65 element is used for the corresponding structural analysis. In the modeling process, shear keys, lifting holes, and prestressing ducts are explicitly included to reflect the actual structural geometry and stress distribution accurately. The midspan and beam-end segments of the segmental box girder contain 34,892 and 51,532 solid elements, respectively, corresponding to 42,665 and 60,334 nodes, respectively. The finite element model of the midspan segment of the segmental box girder is shown in Fig. 3.

### Computational assumptions

Given the numerous factors influencing the temperature and stress fields of precast segmental concrete girders, the following assumptions were made to improve computational efficiency while maintaining acceptable accuracy:

(1) The heat absorbed by reinforcement is neglected in the simulation of the hydration temperature field, and the contribution of ordinary reinforcement is not considered in the structural stress analysis.

(2) After casting, the concrete is assumed to be a continuous, isotropic, and homogeneous material that remains in a linear elastic state under all loading conditions.

(3) The temperature field calculation begins once the segmental concrete of the box girder is entirely cast. It is assumed that no hydration heat is generated during casting and that hydration heat release begins only after casting is complete.

(4) Throughout the simulation of the casting and curing process, the thermal parameters of concrete—including thermal conductivity, specific heat capacity, convection coefficient, and coefficient of thermal expansion—are assumed to remain constant.

(5) The formwork is modeled using spring elements, with corresponding displacement constraints applied to the outer nodes of the springs. After the formwork removal, vertical displacement constraints are imposed on the bottom slab of the segmental girder.

It should be noted that the above modeling assumptions represent a simplification to balance computational efficiency and engineering comparative analysis objectives. The focus of this study is to compare the relative effects of different external conditions on the early-age temperature field of concrete, rather than to accurately predict temperature or stress values. Therefore, maintaining simplified, consistent constitutive relationships for materials helps more clearly reveal the independent effects of external variables, facilitating the determination of maintenance strategies.

### Model parameters

#### Concrete material parameters

The physical parameters of the concrete were determined based on measured data or relevant design specifications during model development. The main parameters are summarized in Table 1.

**Table 1:** Material parameters of concrete.

Material		Elastic Modulus (GPa)	Poisson’s Ratio	Density (kg/m³)	Coefficient of Thermal Expansion (1/K)	Thermal Conductivity (W/(m·K))	Specific Heat Capacity (kJ/kg·°C)
C50 Concrete		35.5	0.2	2450	1 × 10^− 5^	2.94	0.96

During the setting period of concrete, both its elastic modulus and strength vary with time. Therefore, in the simulation analysis of segmental girder casting, the variation of the concrete elastic modulus with time *t* (in days) is expressed as follows:

1 $$E(t)=(1 - {e^{{{( - 0.24 \times t)}^{0.495}}}}) \times 3.55 \times {10^{10}}$$

where *E* is the elastic modulus of concrete, and *t* is the age of concrete (in days).

According to relevant literature, the compressive strength of concrete at the same age is approximately 8 to 10 times greater than its tensile strength during the casting process. Therefore, in this study, the relationship between the tensile and compressive strengths of concrete was determined using the empirical formula proposed by Zhu Bofang^21^.

2 $${R_f}=0.332R_{c}^{{0.6}}$$

3 $${R_c}(t)={R_{c0}}[1+m\ln (t/28)]$$

Where: ${R_f}$ — tensile strength of concrete at age *t*;

${R_c}$— compressive strength of concrete at age *t*;

${R_{c0}}$— 28-day compressive strength of concrete, which is 50 MPa for C50 concrete;

*m*— coefficient related to the type of cement used, taken as 0.1727 in this study.

#### Heat of hydration of cement

The heat of hydration of cement is primarily determined by the cement’s intrinsic properties, including grade, type, and age. In this study, the heat of hydration of cement is calculated using a composite exponential formula:

4 $$Q(\tau )={Q_0}(1 - {e^{ - a{\tau ^b}}})$$

Where: $\tau$ represents age, with the unit being d;

$Q(\tau )$ is the value of the heat of hydration at age $\tau$, and the unit is (kJ/kg);

${Q_0}$ is the final hydration heat as t→∞, and the unit is (kJ/kg).

The concrete for the box girder segments is made of ordinary Portland cement 425^#^, with *Q*_0_=330 kJ/kg, a = 0.69, and b = 0.56.

#### Initial and boundary conditions

(1) Initial Conditions

In general, the initial temperature field can be considered uniformly distributed. For calculating the concrete hydration heat, the casting (placement) temperature of the concrete is taken as the initial temperature.

5 $$T(x,y,z,t)\left| {_{{t=0}}} \right.={T_0}={\mathrm{constant}}$$

(2) Boundary Conditions

When the concrete is exposed to the atmosphere, heat transfer occurs between the surface of the box girder concrete and the surrounding air through convection. Therefore, a third-type (convective) boundary condition is applied in the model. During the structural temperature field calculation, the following thermal boundary conditions were defined according to different curing scenarios:

For natural curing in winter and summer, steel formwork was externally attached to the concrete surface before form removal, and the concrete was directly exposed to air after form removal.

For steam curing in winter, polystyrene foam boards were installed outside the steel formwork, geotextiles were attached to the roof concrete surface, and steam heating was applied before form removal; after form removal, the concrete was directly exposed to air.

For spray curing in summer, steel formwork was attached to the concrete surface externally before form removal, and the concrete was directly exposed to air after form removal, with spray cooling implemented throughout both the pre- and post-demolding periods.

In air, the heat transfer coefficient β\betaβ of the concrete surface primarily depends on the ambient wind speed v or the corresponding wind force level F. The calculation method is expressed as follows:

For smooth surfaces:

6 $$\beta =21.8+13.53v$$

When the surface of the segmental concrete is covered with formwork or an insulation layer, heat is transferred from the concrete surface to the surrounding medium through the insulation layer. In this case, the following equation can be used.

(3) Calculation of the Equivalent Heat Transfer Coefficient

7 $${\beta _s}=\frac{1}{{(1/\beta )+\sum {({h_i}/{\lambda _i})} }}$$

Where: ${\beta _s}$ is the equivalent heat transfer coefficient; $\beta$ is the heat transfer coefficient of the concrete; ${h_i}$ is the thickness of the insulation layer; ${\lambda _i}$ is the thermal conductivity of the insulation layer. Based on the site construction conditions, the annual average wind speed in Xuchang is approximately 2.3 m/s. When a 10 mm steel formwork covers the concrete surface before demolding, the thermal conductivity of the steel plate is $\lambda$= 163.29kJ/($\mathrm{m}\dot{}\; \mathrm{h}\dot{}\: ^\circ\mathrm{C}$). The convective heat transfer coefficient of the steel plate exposed to air is $\beta$= 52.92 kJ/($\mathrm{m}^2\:\dot{}\; \mathrm{h}\dot{}\: ^\circ\mathrm{C}$ ). Therefore, the equivalent heat transfer coefficient of the concrete surface covered with a steel formwork is${\beta _{s1}}=14.65{\mathrm{W/(}}{{\mathrm{m}}^{\mathrm{2}}} \cdot ^\circ {\mathrm{C)}}$. Similarly, the equivalent heat transfer coefficients for concrete covered with a 5 mm geotextile and for concrete covered with 10 mm steel formwork and 10 mm expanded polystyrene (EPS) foam are, respectively, ${\beta _{s2}}=11.87{\mathrm{W/(}}{{\mathrm{m}}^{\mathrm{2}}} \cdot ^\circ {\mathrm{C}})$、${\beta _{s3}}=3.36{\mathrm{W/(}}{{\mathrm{m}}^{\mathrm{2}}} \cdot ^\circ {\mathrm{C)}}$.

During summer spray curing, groundwater at approximately 20 °C was used, with a spray velocity of about 3 m/s. According to basic heat-transfer principles, the average convective heat transfer coefficient hhh during spraying is calculated from Eq. (8):

8 $$h=\frac{{\overline {{N{u_L}}} k}}{L}$$

9 $$\overline {{N{u_L}}} =\left( {0.037\operatorname{Re} _{L}^{{4/5}} - {\mathrm{A}}} \right)P_{r}^{{1/3}}$$

10 $${\operatorname{Re} _L}=\frac{{{u_\infty }L}}{v}$$

Where: $\overline {{N{u_L}}}$ = Nusselt number; k= thermal conductivity of water, taken as 0.599 W/(m·K); *L* = characteristic length of the plate, taken as 2.50 m; ${\operatorname{Re} _L}$ = Reynolds number; ${P_r}$= Prandtl number, taken as 7.02; ${u_\infty }$ = water flow velocity, taken as 3 m/s; *v* = kinematic viscosity of air, taken as 1.006 × 10⁻⁶ m²/s; ${\operatorname{Re} _{x,c}}$ = critical Reynolds number, taken as 5 × 10⁵;${\mathrm{A}}$ = empirical coefficient, taken as 871.

The Reynolds number is 5 × 10^5^ < Re < 10^8^. From this, it is evident that the boundary layer is mixed. According to the above formula, the average convection coefficient during concrete spraying can be calculated as $h=4943.09{\mathrm{W/(}}{{\mathrm{m}}^{\mathrm{2}}} \cdot ^\circ {\mathrm{C}})$.

#### Adiabatic temperature rise calculation

The adiabatic temperature rise is the temperature increase of a concrete element under ideal adiabatic conditions, where no heat is dissipated or lost, and all heat generated by cement hydration is converted into a temperature rise.

The adiabatic temperature rise of concrete can be estimated based on the total heat of cement hydration, as expressed by the following equation:

11 $$\theta (\tau )=\frac{{Q(\tau )W}}{{c\rho }}$$

Where: $Q(\tau )$ is the heat of cement hydration; *W* is cement content; *c* is the specific heat capacity of concrete; $\rho$ is the density of concrete.

#### Heat conduction equation

According to heat conduction theory, the temperature field distribution in a cast-in-place concrete box girder can be regarded as the solution to the heat conduction equation under specified boundary and initial conditions. The heat conduction equation is expressed as follows:

12 $$\frac{{\partial T}}{{\partial t}}=\frac{\lambda }{{\rho c}}(\frac{{{\partial ^2}T}}{{\partial {x^2}}}+\frac{{{\partial ^2}T}}{{\partial {y^2}}}+\frac{{{\partial ^2}T}}{{\partial {z^2}}})+\frac{{\partial \theta }}{{\partial t}}$$

Where: $\lambda$ is thermal conductivity coefficient; *c* is specific heat capacity of concrete, taken as 0.96 kJ/(kg·°C); $\rho$ is density of concrete; $\theta$ is adiabatic temperature rise of concrete.

#### Sensitivity analysis

To more intuitively analyze the cracking risk of concrete during the heat release process of cement hydration under the influence of multiple factors, the sensitivity coefficient (S) is defined as follows:

13 $$S=\frac{{{T_{\hbox{max} }} - {T_{\hbox{min} }}}}{{{T_{\hbox{min} }}}}{{ \times }}100\%$$

where: ${T_{\hbox{max} }}$ represents the maximum peak temperature or thermal stress under a given influencing factor, and${T_{\hbox{min} }}$ represents the corresponding minimum peak temperature or thermal stress.

A higher sensitivity coefficient *S* indicates a more substantial influence of the corresponding factor on the thermal behavior of the precast concrete segment, thereby increasing the risk of cracking during casting.

### Model parameters

**Fig. 4:**
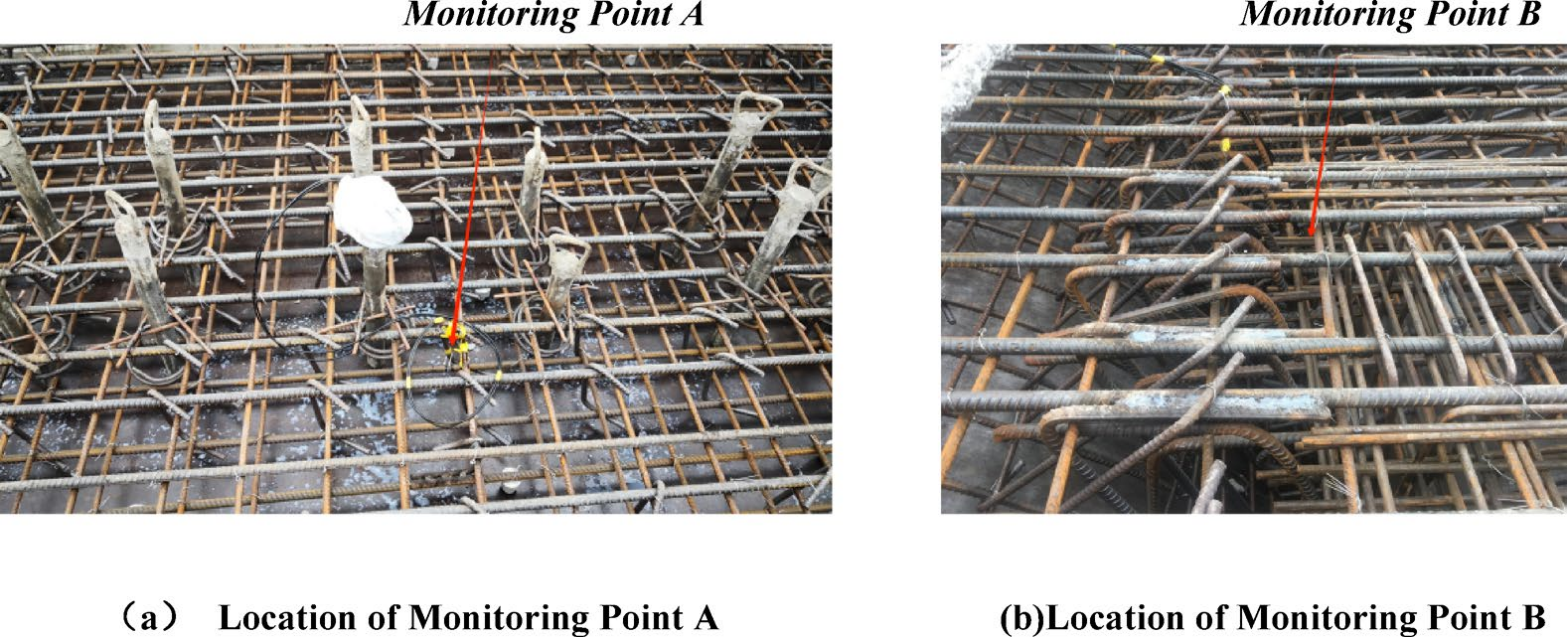
Location of concrete temperature monitoring points A and B.

To verify the accuracy of the FEM in simulating the early-age temperature field of concrete after casting, this section compares model-calculated and measured temperatures at monitoring points on cast segmental beams. For box-section segmental beams, the temperature at the center of the haunch at the junction between the top slab and the web is the highest among the temperatures at other positions. Therefore, this position is selected as the primary monitoring point (Point A), and the center of the top slab is also set as a comparative monitoring point (Point B) for analysis. The layout of the two monitoring points is shown in Fig. 4. Before concrete casting, temperature sensors are fixed on the steel reinforcement near the monitoring points, and continuous monitoring is conducted for 7 days (168 h) afterward.

**Fig. 5:**
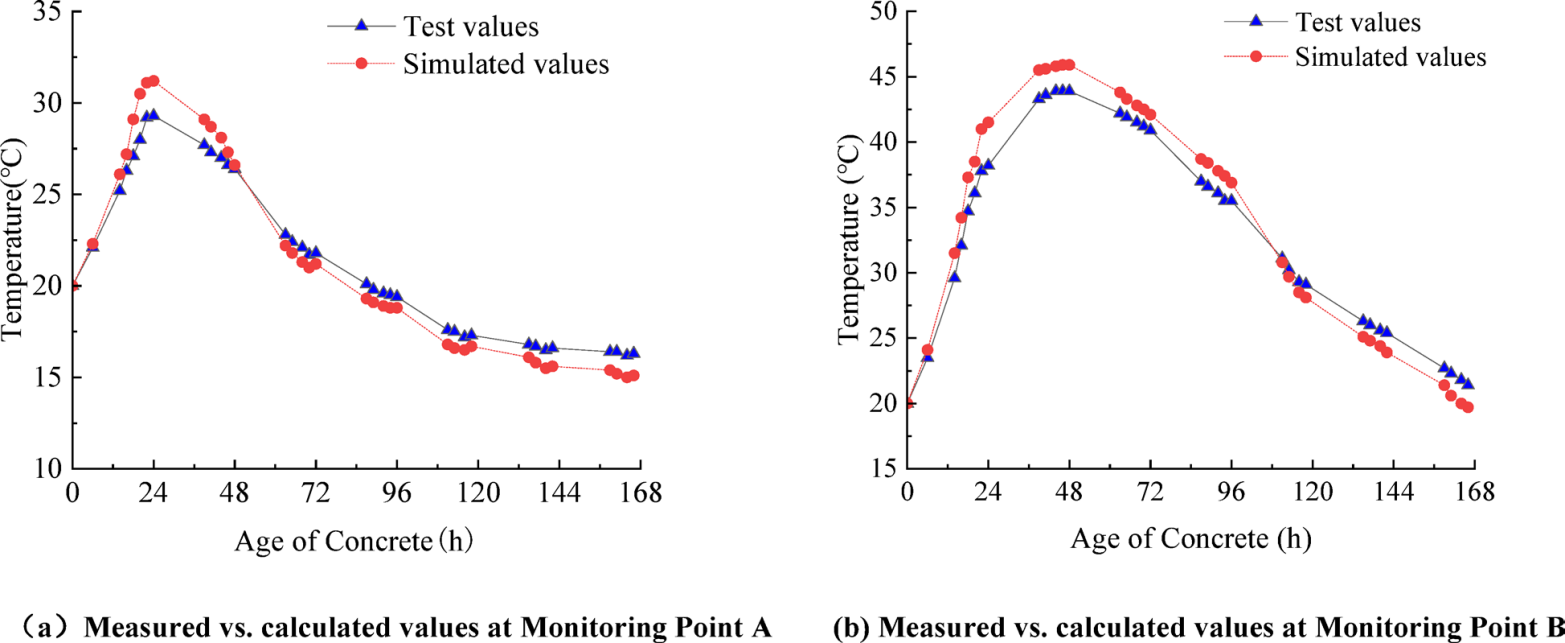
Comparison between simulated temperature and measured temperature of concrete.

Figure 5 presents the comparison curves of the model-calculated and measured concrete temperatures at Points A and B as a function of age. It is observed that the simulated and measured values at both points are generally consistent in their overall variation trends, temperature rise rates, and peak occurrence times. At Point A, the simulated peak temperature is 45.9 °C, while the measured peak temperature is 43.9 °C, resulting in an error of 2 °C and a relative error of 4.6%. At Point B, the simulated peak temperature is 31.2 °C, while the measured peak temperature is 29.3 °C, resulting in an error of 1.9 °C and a relative error of 6.5%. The above comparisons indicate that the model-calculated values at the two monitoring points are close to the measured values, with a temperature error not exceeding 2 °C and a maximum relative error of 6.5%, which meet the engineering accuracy requirements. Moreover, as the concrete temperature increases, the relative error decreases, and the model calculation accuracy improves. The test results confirm that the finite element model established in this study has high computational accuracy and accurately reproduces the variation law of the early-age temperature field of concrete, laying a foundation for subsequent application of the model in the simulation analysis of concrete casting for the Zhengzhou–Xuchang intercity railway segmental beams.

## Multi-factor influence analysis

The heat of hydration in concrete is primarily generated during the early stages after casting. During this period, the cumulative heat released by the hydration reaction raises the core temperature of the cast segment, typically peaking within 24–48 h after pouring. This thermal evolution is significantly affected by several factors, including formwork material, formwork thickness, casting temperature, wind speed, and the presence of prestressing ducts, resulting in fluctuations in the temperature field. At the early stage of casting, concrete’s tensile strength remains relatively low. When the internal–external temperature gradient becomes excessive, thermal cracking is likely to occur. Therefore, to better understand the early-age thermal behavior and potential cracking risks, a multi-factor simulation analysis was conducted for the first seven days (7d) following the casting of the segmental concrete beam under summer meteorological conditions.

### Formwork material

**Fig. 6:**
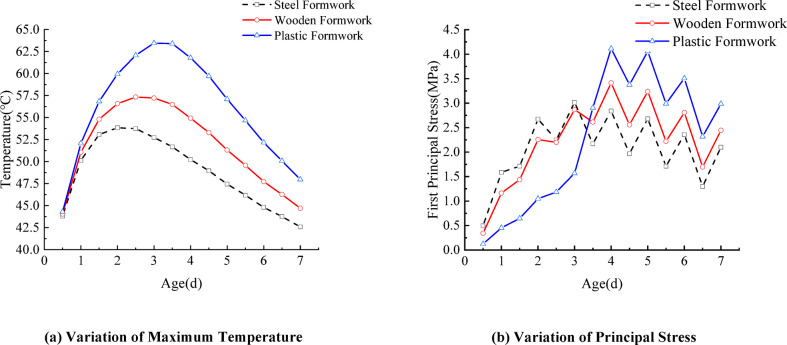
Curves of concrete temperature and stress variation with different formwork materials.

Under identical conditions of formwork thickness (10 mm), placing temperature (20℃), and wind speed (3.0 m/s), three types of formwork materials—steel, timber, and plastic—were analyzed. The corresponding concrete temperature and stress variation curves are shown in Fig. 6. The temperature evolution at the junction between the top slab and the web of the segmental box girder exhibits a similar trend for all formwork types: the internal temperature peaks approximately 2 to 3 days after concrete casting. However, significant differences in peak temperature are observed among different formwork materials. Due to the high convective heat-transfer coefficient and the poor thermal insulation of steel formwork, the concrete reaches its peak temperature on the second day. In contrast, the timber and plastic formworks, with superior insulation properties, delay the temperature peak to about 2.5 days and 3 days, respectively. The maximum temperature difference between the steel and plastic formworks reaches 17.8%. Before demolding, the segment cast with plastic formwork experiences the lowest stress. After demolding, however, the stress in the plastic formwork specimen increases sharply. This occurs because the plastic formwork’s strong insulation reduces surface heat loss before demolding, resulting in a smaller internal–external temperature difference. Once the formwork is removed, rapid surface cooling causes a sharp drop in temperature and a significant temperature gradient, leading to a rapid rise in tensile stress. Therefore, the better the thermal insulation performance of the formwork material, the later the temperature peak occurs and the higher the peak temperature and post-demolding thermal stress become. The maximum difference in concrete temperature stress between the steel and plastic formworks reaches 36.1%. Plastic formwork, with its excellent thermal insulation, reduces heat loss from the concrete surface before form removal and effectively controls early-age temperature stress. However, after form removal, the sudden loss of insulation causes a rapid drop in the concrete surface temperature, potentially creating a large temperature difference between the interior and exterior, thereby increasing the risk of cracking. Therefore, when removing formwork from concrete, it is necessary not only to verify that the concrete strength meets specification requirements and to use steel formwork with good heat dissipation, but also to ensure that the concrete temperature does not exceed the allowable limit during form removal. If the temperature is too high, rapid cooling will cause a large temperature difference between the interior and exterior, which may lead to concrete cracking.

### Formwork thickness

**Fig. 7:**
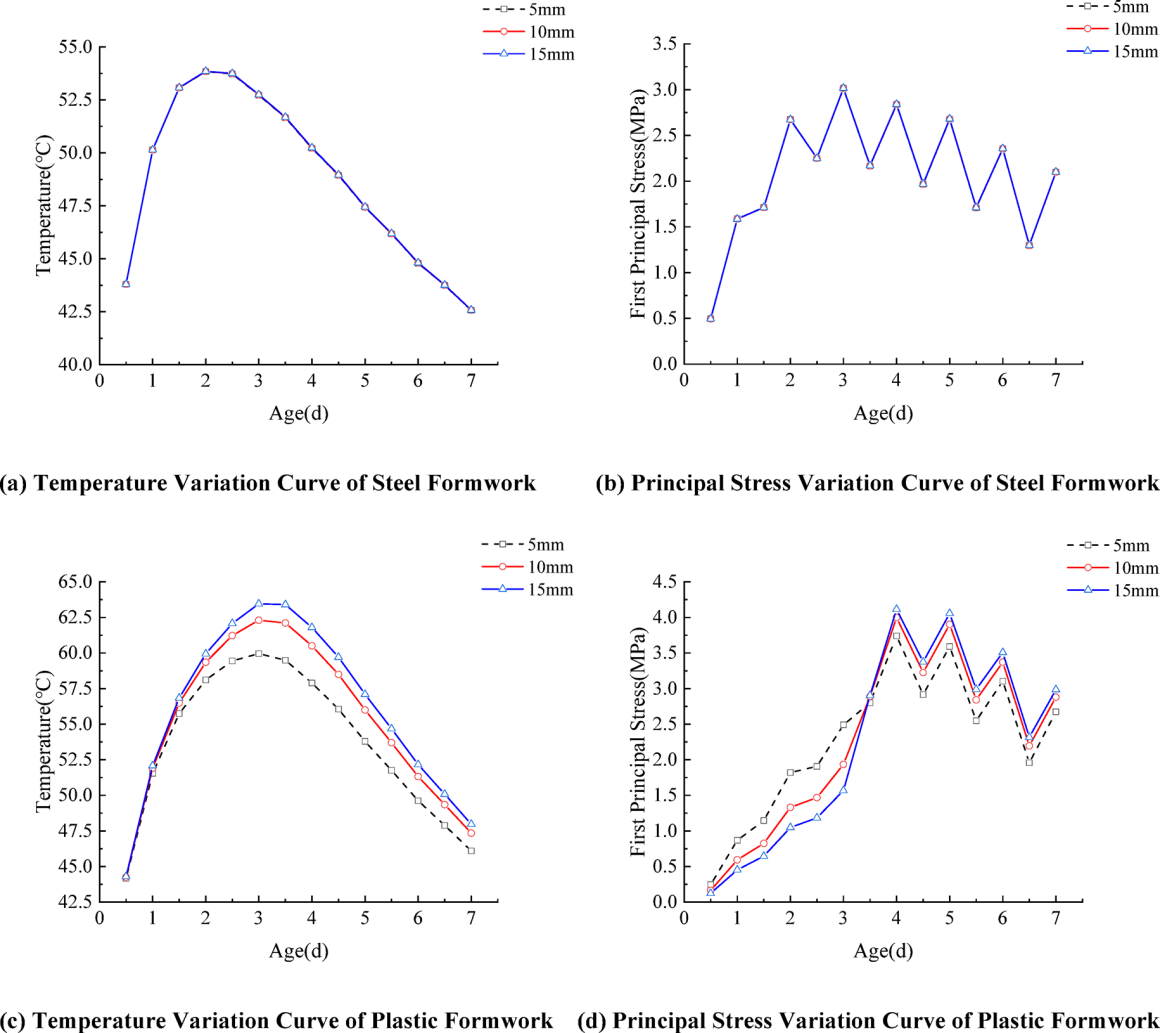
Concrete temperature and stress variation curves for different formwork materials and thicknesses.

Under identical conditions of formwork material, placing temperature (20℃), and wind speed (3.0 m/s), three formwork thicknesses—5 mm, 10 mm, and 15 mm—were analyzed, based on the commonly used formwork thicknesses in current railway precast beam yards. The corresponding temperature and stress variation curves of the segmental concrete girders are shown in Fig. 7. For steel formwork, the maximum and minimum peak temperatures in the 5 mm, 10 mm, and 15 mm cases are nearly identical. Variations in steel formwork thickness within the typical engineering range (5–15 mm) exert minimal influence on the early-age temperature field of concrete box girders. This phenomenon is primarily attributed to steel’s high thermal conductivity and poor insulation properties, with adjustments to plate thickness having little effect on heat transfer. Consequently, such variations result in negligible changes to both the temperature field and the development of thermal stress in box girder concrete.

The impact of plastic formwork thickness must be analyzed from two perspectives: thermal inertia and thermal insulation performance. As illustrated in Fig. 7(c), increasing the plastic formwork thickness from 5 mm to 15 mm increases the peak temperature in the concrete core by only approximately 3 °C, indicating limited improvement in insulation capacity. However, its influence on temperature evolution is more critical. Thicker formwork exhibits greater thermal inertia, leading to slower heat dissipation and a more gradual temperature rise curve during the heating phase. During the cooling period after formwork removal, when surface temperatures drop sharply, the concrete core under thicker formwork retains higher temperatures, resulting in a larger temperature gradient between the interior and exterior immediately after demolding.

Therefore, the key consideration in selecting plastic formwork thickness is not controlling the absolute maximum temperature, but rather regulating the rate of temperature change before and after formwork removal and the associated rapid stress increase. In contrast, due to steel’s extremely high thermal conductivity, variations in thickness within the 5–15 mm range have almost no effect on thermal inertia, resulting in nearly overlapping temperature and stress curves for concrete with steel formwork of different thicknesses.

### Wind speed

**Fig. 8:**
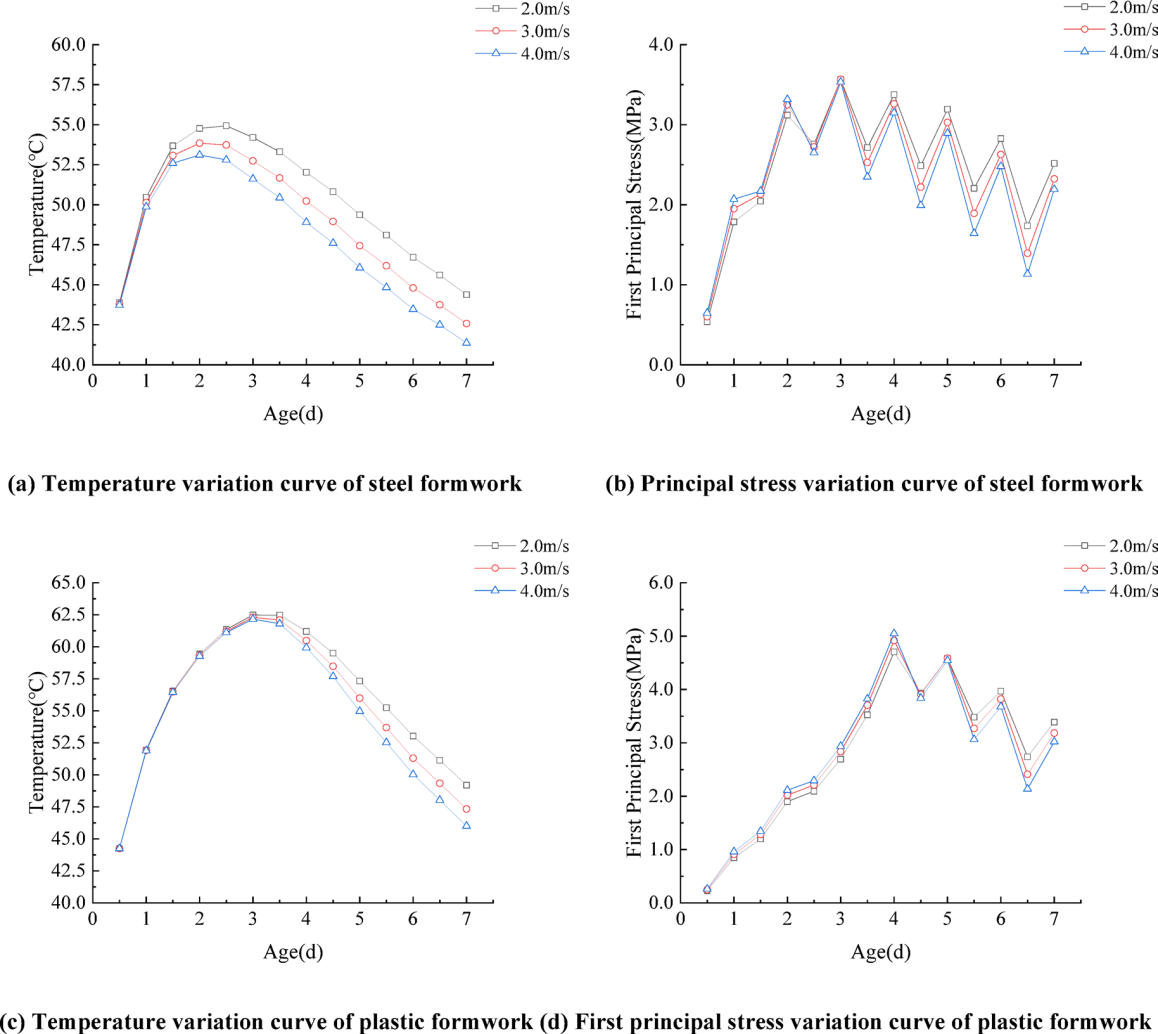
Temperature and stress variation curves under different wind speeds.

Based on long-term meteorological monitoring data from the Xuchang Meteorological Administration, the local annual mean wind speed is approximately 3.0 m/s. Accordingly, the research scope for typical ambient wind speeds was defined as 2.0–4.0 m/s. Systematic investigations were conducted to examine the evolution of the temperature and stress fields in concrete box-girder segments at wind speeds of 2.0 m/s, 3.0 m/s, and 4.0 m/s. All tests were performed under controlled conditions, including a consistent concrete placement temperature of 20 °C, identical formwork materials, and a uniform formwork thickness of 10 mm. Comparative curves illustrating temperature and stress variations across different wind speed conditions are presented in Fig. 8. The temperature variation at the junction between the top and web slabs shows a consistent trend across different wind speeds. As wind speed increases, the surface heat loss from concrete accelerates, leading to a faster temperature drop and a larger internal–external temperature gradient. Consequently, the peak temperature decreases, and its occurrence time advances by approximately 0.5 days. Due to the excellent thermal insulation of plastic formwork, surface heat dissipation is limited, and the temperature at the junction between the top and web slabs is less affected by external conditions. As a result, the temperature variation curves under the three wind speed conditions are nearly identical. The maximum stress in the concrete box girder initially increases and then decreases with age. For steel and plastic formworks, the maximum stress peaks occur around the third and fourth days, respectively. Before demolding, the maximum stress at 4.0 m/s is higher than at the other two wind speeds. However, after demolding, the maximum stress becomes lower. This occurs because during early-age hydration, higher wind speeds accelerate surface heat dissipation, thereby increasing the temperature gradient and, in turn, the thermal stress. After demolding, the surface releases more heat at high wind speeds, resulting in smaller internal–external temperature differences and reduced thermal stress. Therefore, during early-stage demolding of precast concrete box girders, attention should be paid to wind speed conditions. When necessary, wind-shielding measures should be implemented to reduce rapid heat loss, minimize internal–external temperature differences, and lower the risk of thermal cracking in the concrete.

### Casting temperature

**Fig. 9:**
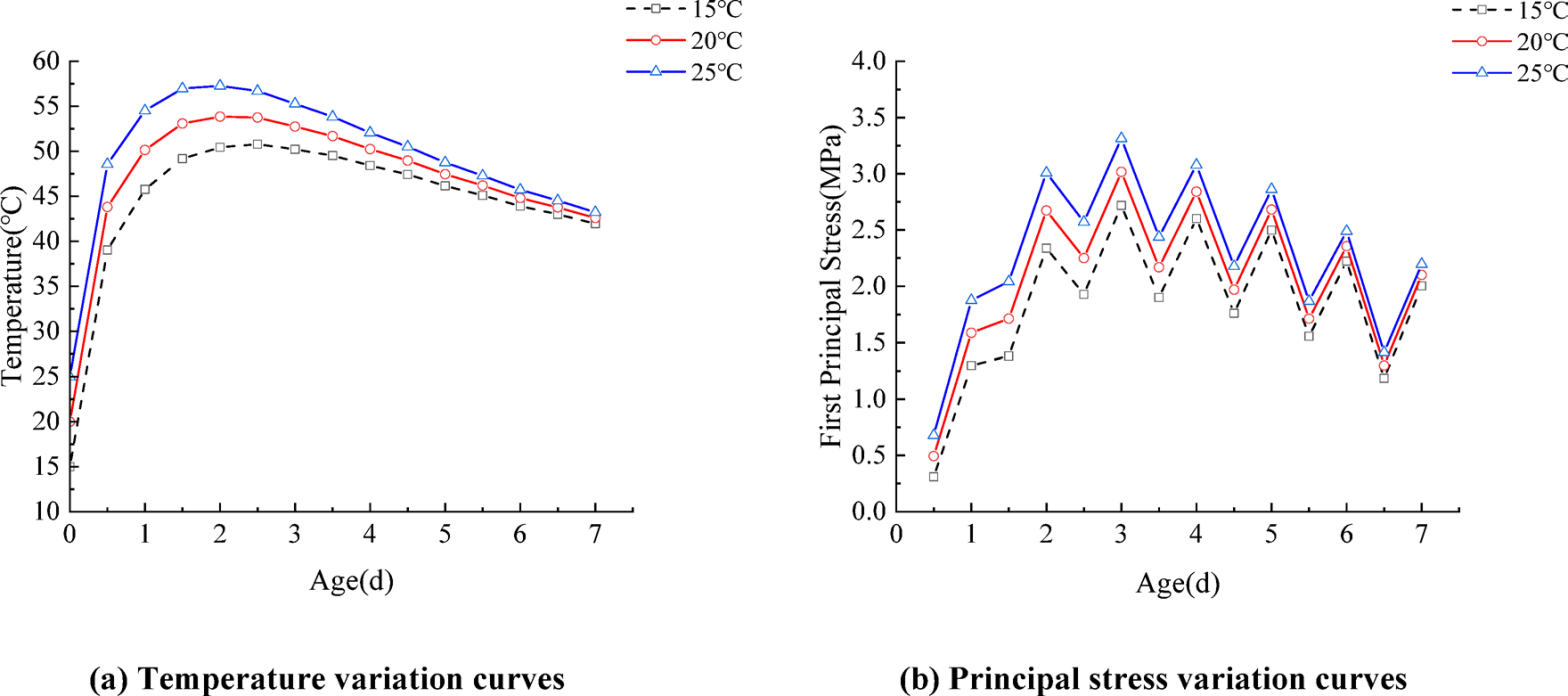
Temperature and stress variation curves under different casting temperatures.

The casting temperature refers to the average temperature of the water, cement, sand, and other aggregates at the time of concrete placement. According to the *Concrete Quality Control Standard*, the casting temperature should be higher than 5.0 °C during winter concreting and lower than 35.0 °C during summer construction. In engineering practice, for critical infrastructure projects and concrete structures requiring high long-term durability, the concrete placement temperature is typically controlled within 15.0 °C to 25.0 °C to optimize material performance. Considering that the annual average ambient temperature at the project site is approximately 15 °C and that the Standard for Construction of Mass Concrete stipulates that the placement temperature should not exceed 25 °C, the experimental placement temperature range was defined as 15.0 °C to 25.0 °C. Three discrete values of 15.0 °C, 20.0 °C, and 25.0 °C were selected for systematic investigation. Under controlled conditions, including a constant wind speed of 3.0 m/s, steel formwork, and a formwork thickness of 10 mm, the early-age temperature and stress response curves of the box girder segment concrete are illustrated in Fig. 8. As shown in Fig. 9(a), when the casting temperature is 15.0 °C, the peak temperature occurs at approximately 2.5 days after casting and is the lowest among the three cases.

For casting temperatures of 20.0 °C and 25.0 °C, the peak temperatures occur around 1.5 days and 2 days, respectively. As the casting temperature increases from 15.0 °C to 25.0 °C, the peak temperature at the junction of the top slab and the web increases from 50.8 °C to 57.3 °C—an increase of 12.7%. This indicates that the maximum temperature of the box girder is positively correlated with the casting temperature, and higher casting temperatures lead to earlier peak occurrences. Before the hydration reaction begins, the concrete temperature equals the casting temperature. According to the law of energy conservation, the peak temperature equals the sum of the temperature rise due to hydration and the initial casting temperature, minus the heat loss. The earlier peak temperature is attributed to the fact that the rate of cement hydration is directly influenced by the casting temperature—the higher the casting temperature, the faster the temperature rises due to the heat of hydration. As shown in Fig. 9(b), at different casting temperatures, the maximum stress in the segmental girder increases with age, reaches a peak on the third day, and then gradually decreases. When the casting temperature rises from 15.0 °C to 25.0 °C, the peak temperature stress increases by 21.7%. This demonstrates that higher casting temperatures lead to greater temperature-induced stress because higher initial temperatures cause greater hydration heat release and a larger temperature gradient, thereby resulting in higher thermal stress levels.

### Prestressing ducts

**Fig. 10:**
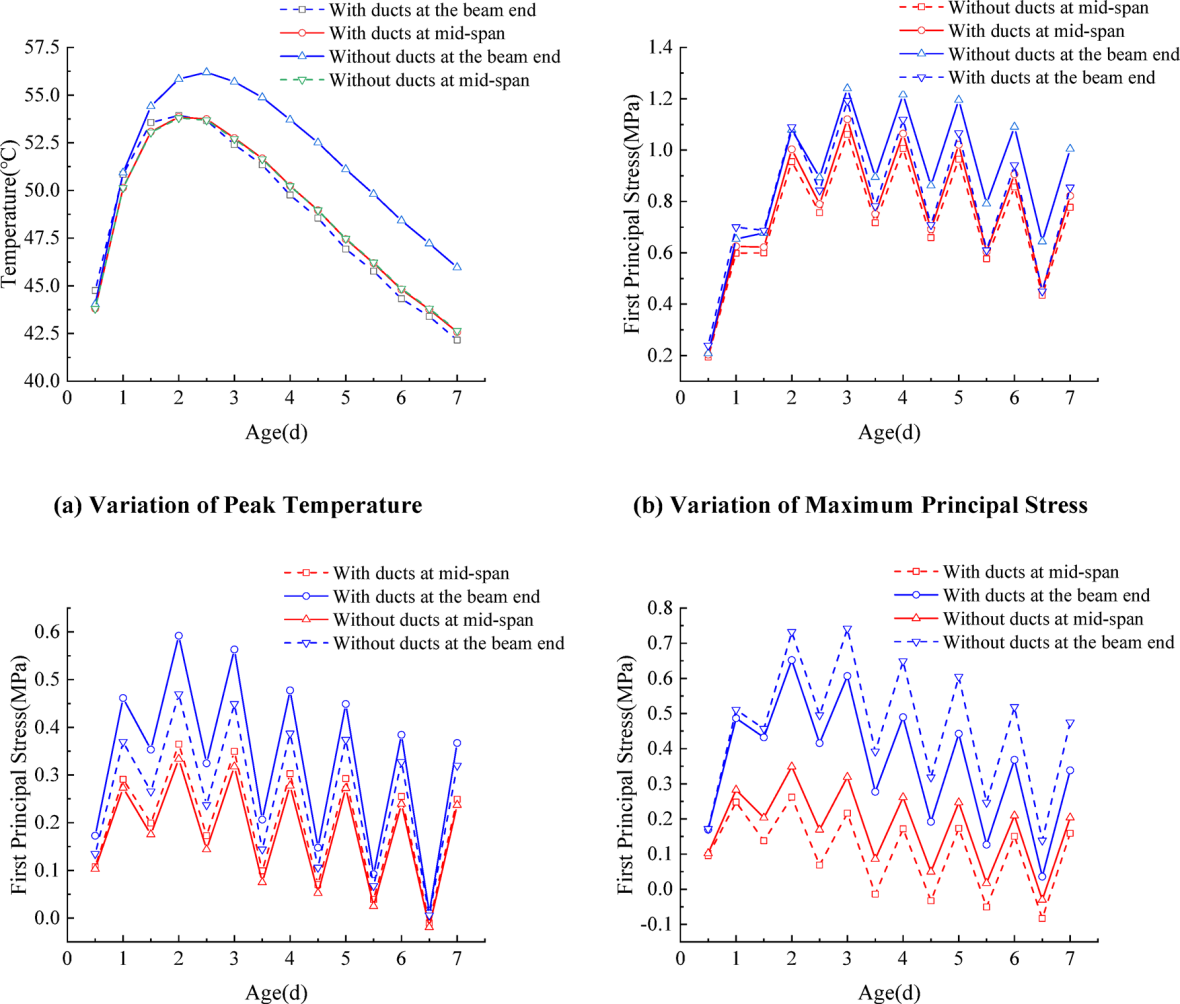
Temperature and stress variation curves of segmental girders with and without prestressing ducts.

Prestressing ducts were arranged in both mid-span and end segments of the box girder. For the end segments, ducts were placed in the webs and at the junction between the top slab and the web. In contrast, for the mid-span segments, the ducts were mainly located in the lower portion of the web and along the bottom slab. The temperature and stress variation curves for segments with and without prestressing ducts are shown in Fig. 10(a) and (b), respectively. The prestressing ducts exerted a relatively minor influence on the peak temperature of the mid-span segment but had a more pronounced effect on the end segment. The peak temperature of the end segment with ducts was approximately 4.3% lower than that of the segment without ducts, and the peak occurred about 0.5 days earlier. Similarly, the peak thermal stress in the end segment with ducts was about 4.2% lower than in the one without ducts. This can be attributed to the ducts located at the junction between the top slab and the web in the end segment, which facilitated heat dissipation during hydration. Consequently, the internal peak temperature decreased, and the time to reach it was shortened.

In contrast, for the mid-span segment, since the prestressing ducts were primarily located along the lower web and bottom slab—rather than near the junction between the top slab and the web—their effect on the temperature gradient between the inner and outer concrete was minimal. Therefore, the peak temperature and thermal stress of the mid-span segment did not vary significantly. As shown in Fig. 10(c) and (d), the temperature stress in the top and bottom slabs of the segmental girder with ducts was noticeably lower than that in segments without ducts. This further confirms that the inclusion of prestressing ducts effectively reduces the internal–external temperature differential, thereby lowering thermal stress and minimizing the risk of cracking in precast segmental concrete girders.

### Multi-factor sensitivity analysis

**Fig. 11:**
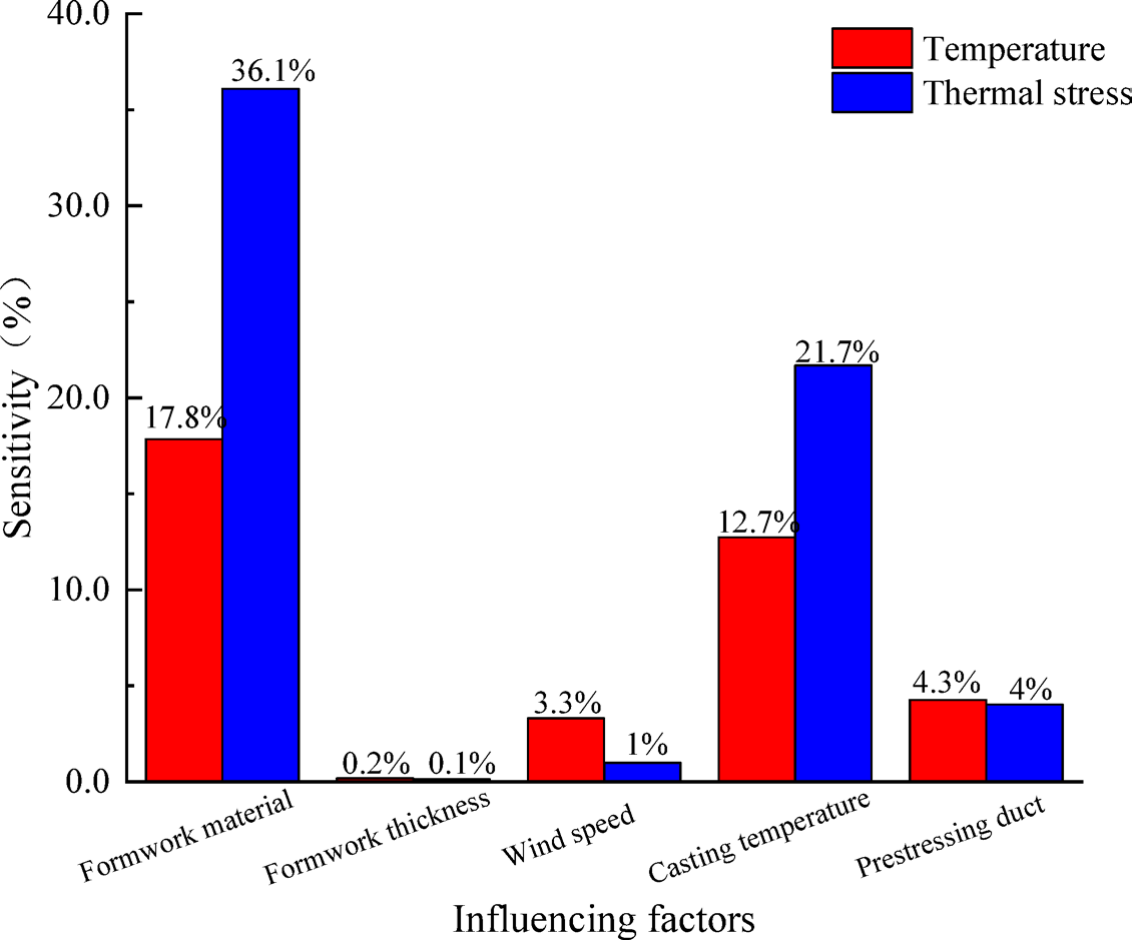
Sensitivity analysis of multiple influencing factors.

Based on the above analysis, the effects of curing ambient temperature, formwork material, formwork thickness, casting temperature, ambient wind speed, and the presence of prestressing ducts on the thermo-mechanical coupled stress of segmental girders were investigated. The calculated sensitivities of these influencing factors to the peak temperature and thermal stress are shown in Fig. 11. It can be observed that the formwork material and casting temperature have the most significant influence on the hydration heat-induced thermal effects in the precast concrete segmental girder. The sensitivities of temperature and thermal stress to the formwork material are 17.8% and 36.1%, respectively. Therefore, it is recommended to adopt steel formwork with good thermal conductivity for precast segmental girders to facilitate effective heat dissipation. The effects of wind speed and prestressing ducts are relatively minor, with sensitivities of both temperature and thermal stress remaining within 5%. In addition, the thickness of the steel formwork has an almost negligible influence on the hydration heat temperature effect, with sensitivities of temperature and thermal stress approaching zero. Hence, when using steel formwork, its thickness needs to meet the structural support requirements.

## Refined curing temperature field and thermal stress analysis

### Refined curing control measures

To delay the peak temperature, reduce temperature gradients, and achieve more precise control of curing temperature, a series of precision curing measures was developed based on results from a multi-factor sensitivity analysis, accounting for the effects of formwork material, casting temperature, prestressing ducts, and wind speed.

**Fig. 12:**
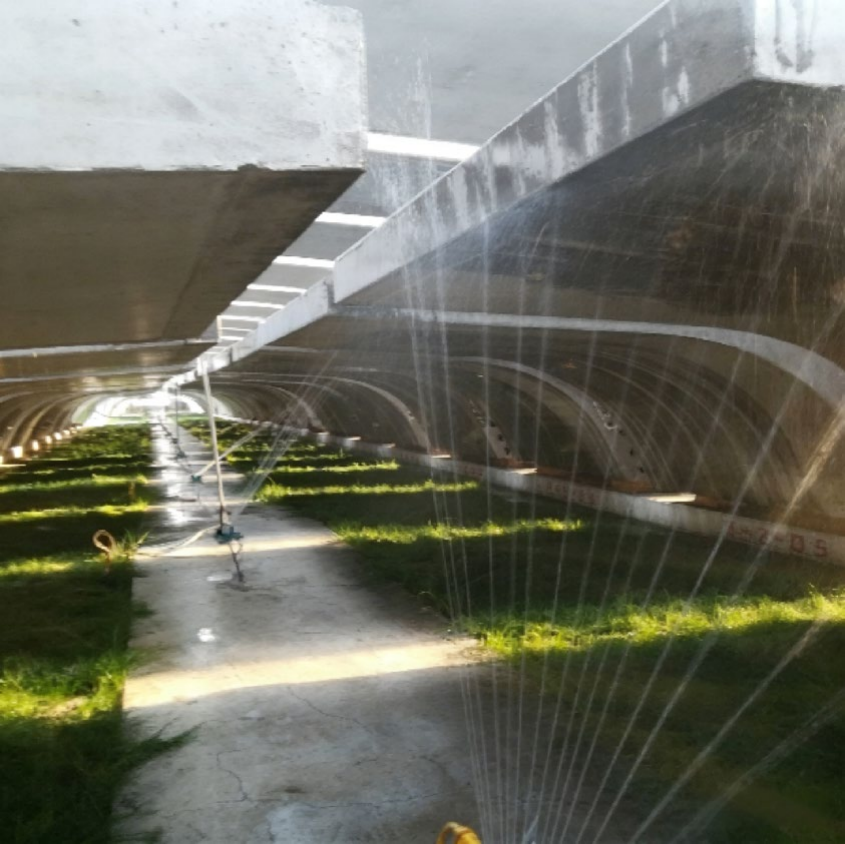
Intelligent automatic sprinkling curing system.

During the summer season, an intelligent spraying system is employed for concrete curing, as shown in Fig. 12. The casting duration for each full-span segmental beam does not exceed 3 h. Formwork removal is carried out when the concrete is 3 days old. Before formwork removal, the concrete surface is covered with a steel formwork in contact with the air, while after formwork removal, the concrete surface is directly exposed to the atmosphere. Immediately after casting, high-pressure water spraying is applied using water jets at 0.3–0.5 MPa and 2–4 m/s. Each spraying cycle lasts 10–15 min, and water cooling is performed both before and after formwork removal. The curing water is qualified groundwater, with a stable annual temperature of approximately 15–17 °C. The total curing time is at least 30 days.

**Fig. 13:**
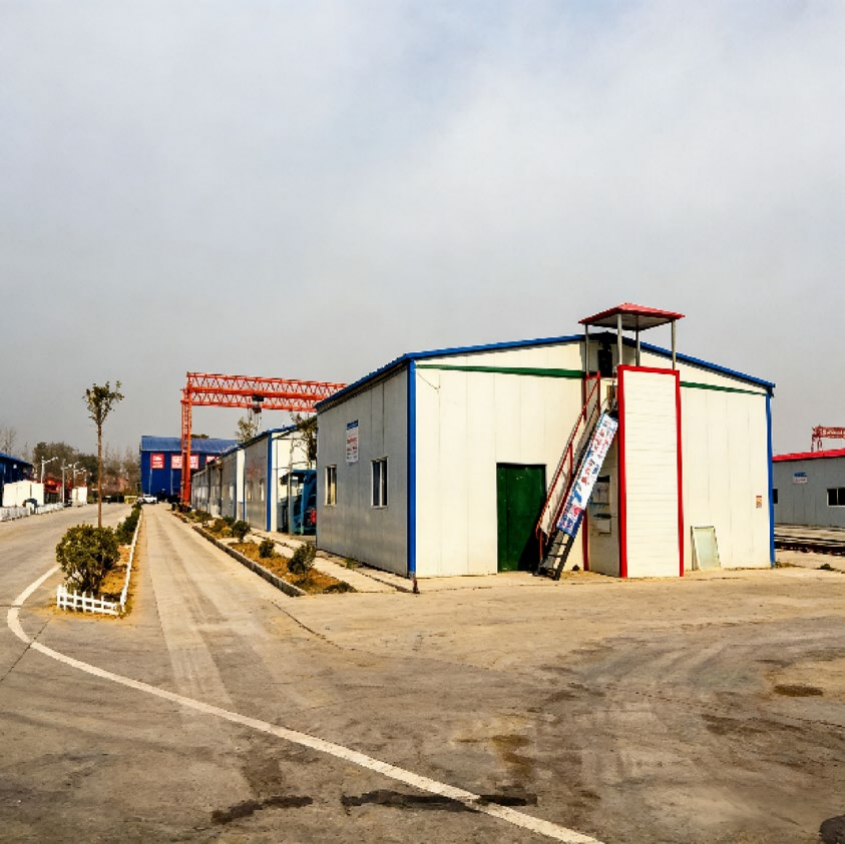
Adjustable intelligent steam curing shed.

During the winter season, steam curing is conducted in an intelligent curing chamber, as illustrated in Fig. 13. Because the ambient temperature in Xuchang typically falls below 5 °C, the curing process uses a fireproof, rock-wool-insulated steel chamber with steam heating. The casting duration for each full-span segmental beam does not exceed 3 h. Steam curing begins approximately 4 h after casting and continues until formwork removal at 3 days of age. Before formwork removal, polystyrene foam boards are attached to the exterior of the steel formwork, while geotextile fabric is applied to the top slab surface, both in contact with the air and heated by steam. After formwork removal, the concrete is directly exposed to the atmosphere. The steam-curing process consists of four stages: preheating (holding), heating, constant-temperature, and cooling. During preheating, the curing chamber’s internal temperature is maintained at 10 °C. The heating stage begins 4–6 h after casting, with a maximum rate of 10 °C/h. Once the curing temperature reaches 45 °C, the process enters the constant-temperature stage, which lasts for 12 h. The cooling stage follows, with a cooling rate limited to 10 °C/h. After steam curing is complete, since the ambient winter temperature remains below 5 °C, additional thermal insulation is applied to the beam surfaces, and the chamber temperature is maintained at approximately 10 °C to ensure adequate curing.

### Temperature field

**Fig. 14:**
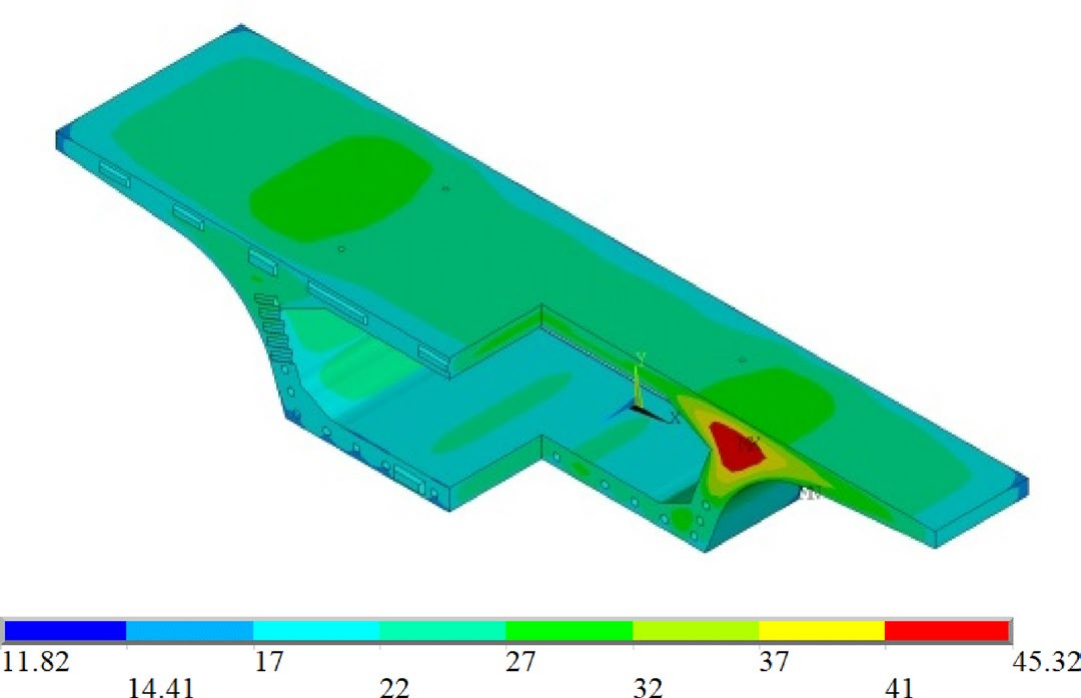
Temperature field distribution.

**Fig. 15:**
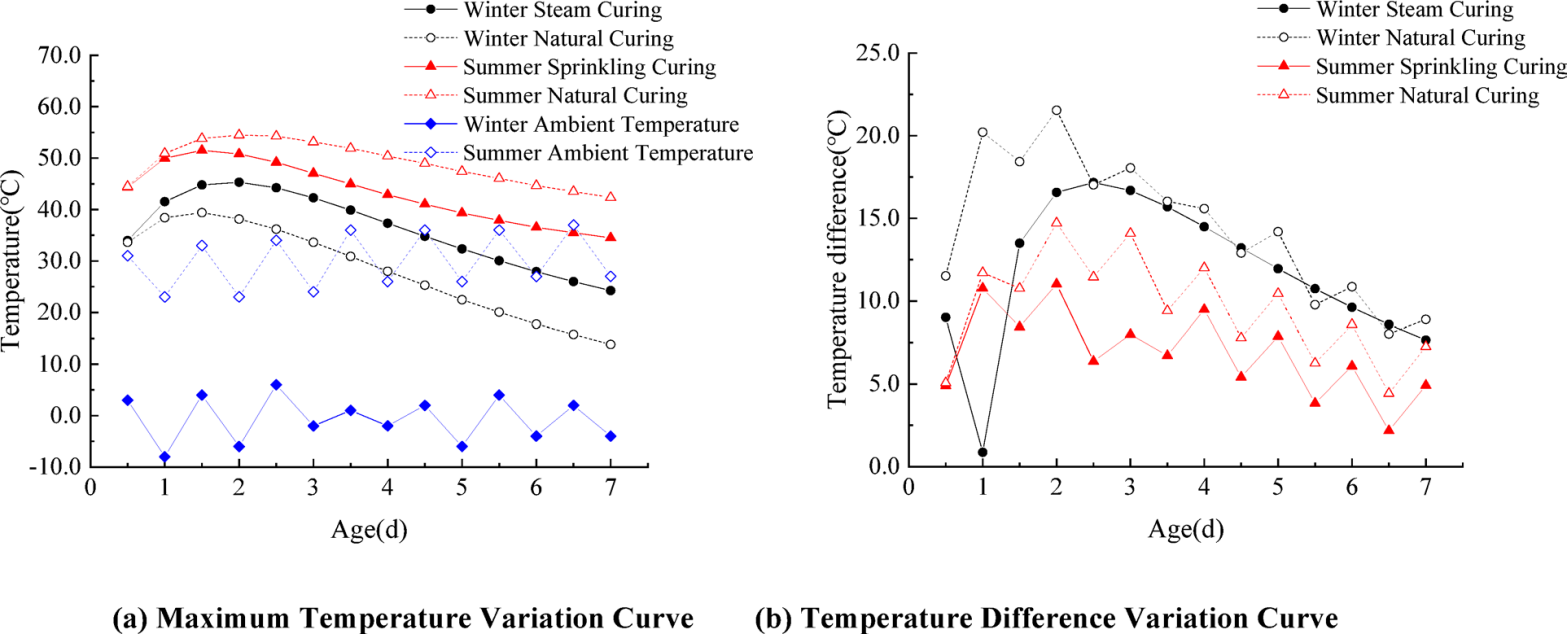
Maximum temperature and temperature difference variation curves.

For the temperature field analysis, the simulation used a time step of 0.5 days over 14 steps, modeling both the mid-span and end segments of the beam under extreme winter and summer climatic conditions. The simulated temperature field distributions, peak temperatures, and temperature difference curves are shown in Figs. 14 and 15.

The results indicate that under different curing conditions, the temperature field distributions of the segmental beams in both winter and summer follow similar patterns, exhibiting symmetry in both longitudinal and transverse directions. The temperature within the box-girder segments experiences two distinct stages with concrete age: a heating phase followed by a cooling phase. Due to the large volume and limited heat dissipation capacity of the concrete at the junction between the top slab and the web, the high-temperature zone is concentrated in this core region. Because the top and bottom slabs of the box girder are relatively thin, heat transfer is rapid, resulting in a smaller internal–external temperature difference. By the seventh day, the concrete’s internal temperature nearly matches the ambient temperature. Steam curing during winter effectively delays the peak temperature and reduces the internal–external temperature difference, thereby mitigating the risk of thermal cracking. In contrast, summer spray curing cools the concrete surface by misting water, accelerating heat dissipation, reducing the temperature gradient, and lowering the likelihood of cracking.

### Thermal stress

After numerically analyzing the thermal effects of hydration heat in the segmental beam, the computed temperature field was applied as a load to the structural stress analysis model to perform a thermo-mechanical coupled stress analysis. This study evaluates the relative risk of early cracking by comparing the calculated temperature-stress with the concrete’s tensile strength at the same age. In fact, concrete cracking criteria typically need to consider factors such as the degree of constraint, strain capacity, and creep. Since the main purpose of this study is to verify that refined curing can effectively reduce the temperature difference between the internal and external concrete and temperature stress, reduce the risk of concrete cracking, and ensure the quality of concrete segmental beams by comparing the effects of refined curing control measures and natural curing on the temperature field and stress field of concrete, rather than focusing on the establishment of concrete cracking criteria, a simple method of comparing concrete stress with the tensile strength of concrete at the same period is used in this paper to judge the risk of concrete cracking.

**Fig. 16:**
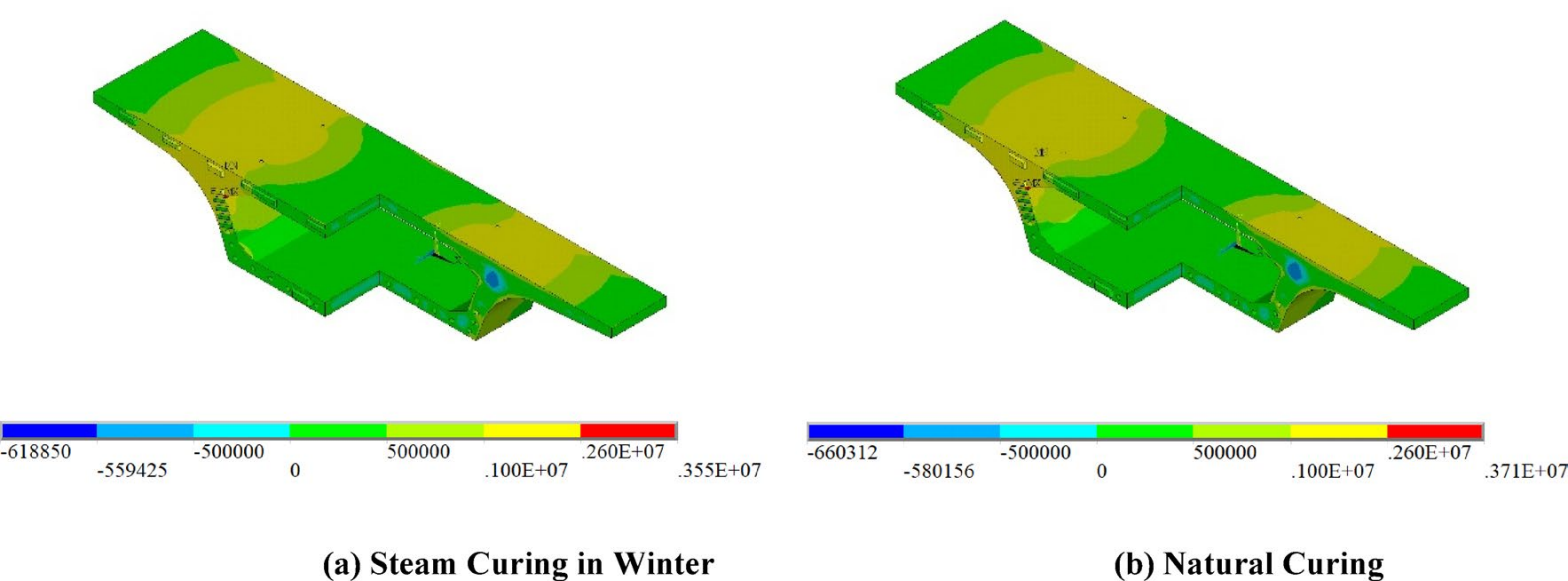
Temperature stress distribution.

The study found that, after concrete is poured in segmental beams, thermal stress is related to the temperature gradient. The temperature changes are severe during winter construction, and the cracking risk for segmental beams is higher. Figure 16 shows the temperature stress distribution during winter steam curing and natural curing.

**Fig. 17:**
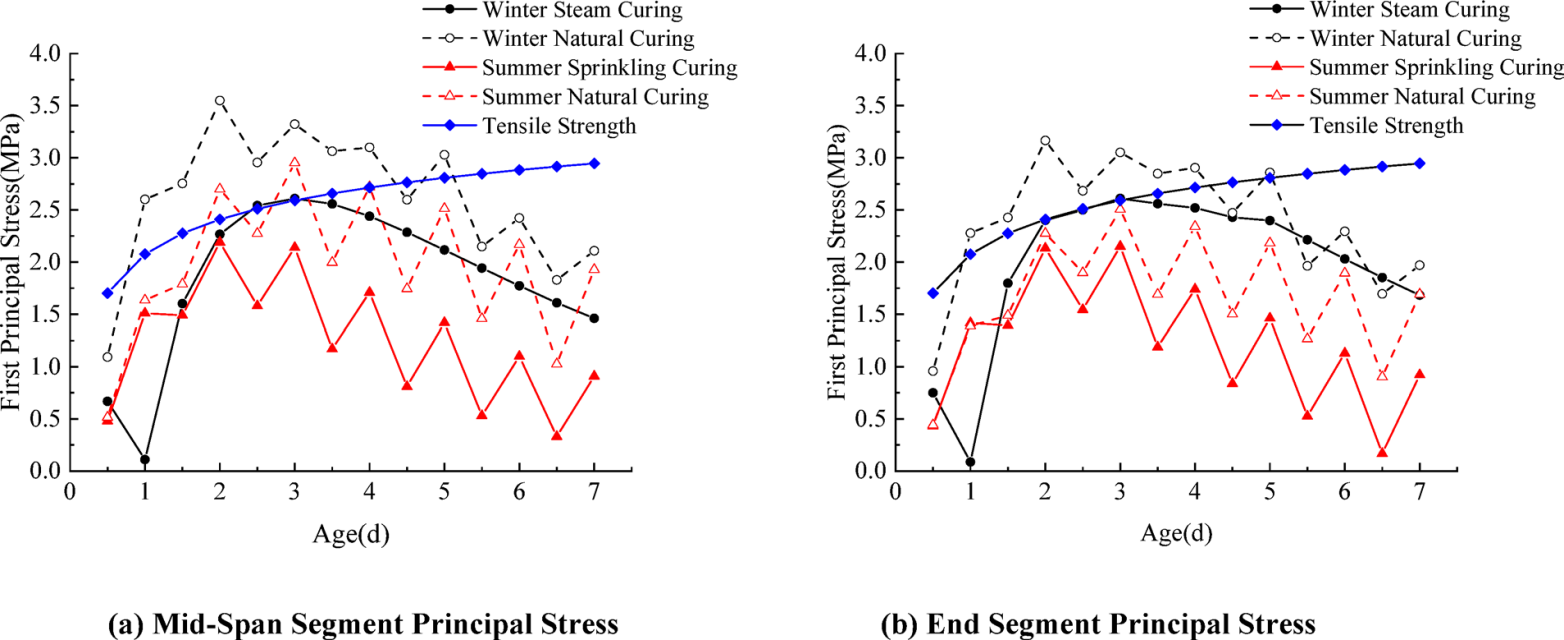
Maximum principal stress variation curves.

The temperature stress distribution is shown in Fig. 16. In the early stage after concrete casting, a large amount of heat is generated by cement hydration. Because heat transfer within the concrete is slow, significant heat accumulates within the segment, resulting in relatively high internal temperatures. Meanwhile, the outer surface dissipates heat more rapidly through convection with the surrounding air, leading to a lower surface temperature. This creates a substantial internal–external temperature gradient: the outer surface contracts as it cools, while the inner core expands as it remains warm. The mutual restraint between these deformations generates compressive stress within the interior and tensile stress at the surface. The overall stress distribution pattern of the segmental beam is similar in both winter and summer. The maximum tensile stress is concentrated at the junction between the top slab and the web haunch, where structural complexity induces local stress concentration. Due to lower ambient temperatures in winter, the temperature gradient between the inner and outer surfaces of the box girder is more pronounced, resulting in significantly higher thermal stresses than those observed under summer conditions.

The variation of maximum stress is illustrated in Fig. 17. Under winter conditions, the maximum tensile stress of the segmental beam occurs on Day 2 for natural curing and on Day 3 for steam curing. For natural curing, the maximum tensile stresses at the midspan and beam-end segments are 3.6 MPa and 3.2 MPa, respectively, which are 1.50 times and 1.23 times the tensile strength of concrete at the same age, indicating a relatively high cracking risk. In contrast, under steam curing, the maximum tensile stress at both locations is about 2.5 MPa, which is below the concrete’s tensile strength at the same age, suggesting a relatively low risk of cracking. Winter steam curing reduces peak temperature-stress in the mid-span and beam-end segments by 30.6% and 21.9%, respectively, and can delay the occurrence of the maximum tensile stress by about 1 day. During summer, the maximum tensile stresses occur on Day 3 under natural curing and on Day 2 under spray curing. For natural curing, the maximum tensile stresses at the midspan and beam-end segments are 3.0 MPa and 2.5 MPa, respectively, with the beam-end value exceeding the concrete’s tensile strength. The maximum tensile stress at the beam end exceeds 1.15 times the concrete tensile strength, indicating a relatively high risk of cracking. The maximum values at the mid-span and beam-end segments of the latter are 2.2 MPa and 2.1 MPa, respectively, both of which are lower than the tensile strength of concrete, indicating a relatively low cracking risk. Summer spray curing reduces the peak temperature stress of the mid-span segment and beam-end segment by 26.7% and 16.0%, respectively. The evolution of peak temperature and temperature difference follows a similar pattern in both winter and summer, reaching their maxima around Day 2.

In contrast, the maximum tensile stress occurs approximately one day later (Day 3). This indicates a distinct time lag between the peak temperature gradient and the resulting thermal stress. Overall, the results confirm that steam curing in winter and spray curing in summer are both reasonable and adequate curing strategies for minimizing thermal cracking in segmental beams.

**Fig. 18:**
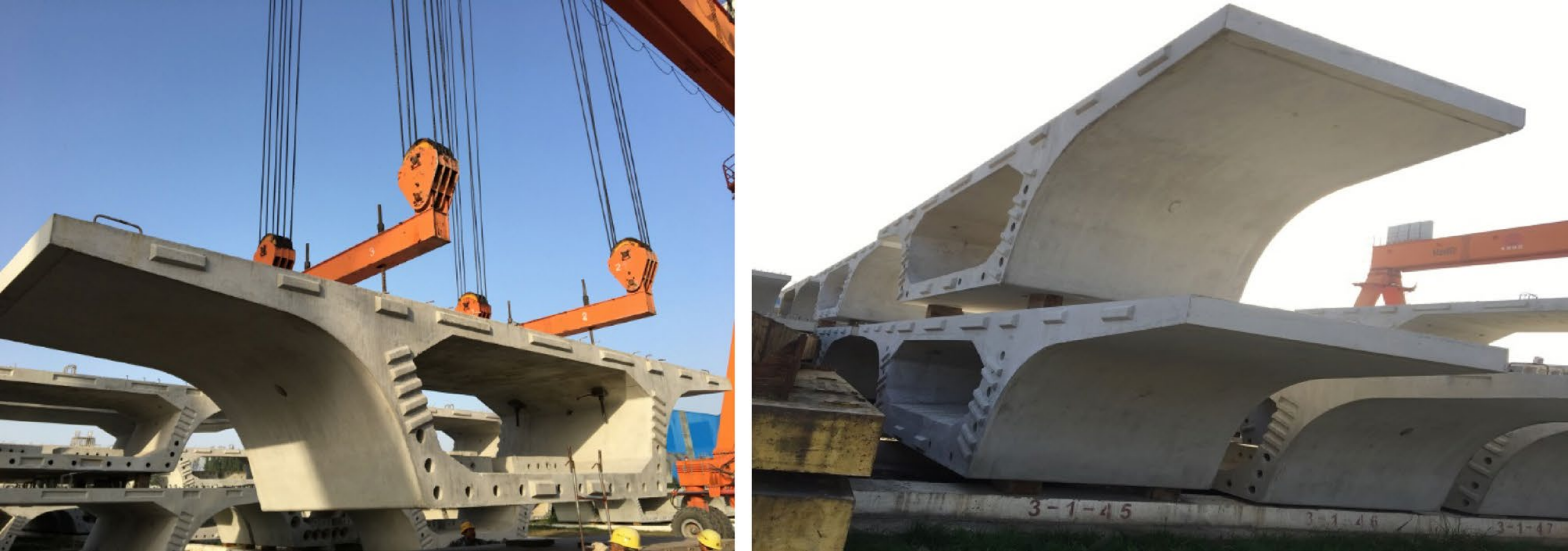
Exterior quality of the segmental beam.

By implementing refined curing measures, including an intelligent spray-curing system in summer and an intelligent steam-curing chamber in winter, the risk of concrete cracking is effectively reduced. As shown in Fig. 18, the exterior quality of the precast segmental beams is excellent—no visible cracks, voids, or other defects were observed. This confirms the rationality and scientific validity of the refined curing scheme.

## Conclusions

To investigate the hydration heat temperature field of precast concrete segmental beams in intercity railway bridges and improve the quality of segment fabrication, this study analyzed the key factors influencing the casting and curing processes of precast concrete box-girder segments. Considering the local climatic conditions of the precast yard, this study examined the effects of curing ambient temperature, formwork material, formwork thickness, casting temperature, wind speed, and prestressing duct layout on the thermal–structural coupling stress of the precast concrete segmental box girder. Based on these findings on key influencing factors, a refined curing control scheme for precast concrete segmental box girders was developed. The main conclusions of this study are as follows:

(1) The formwork material and casting temperature significantly affect the hydration heat temperature characteristics of precast concrete segmental box girders. Among these two factors, the formwork material has the greatest influence, with maximum temperature and thermal stress sensitivities of 17.8% and 36.1%, respectively. In contrast, wind speed and the layout of prestressing ducts have relatively minor effects, with both their temperature and thermal stress sensitivities below 5%. The thickness of steel formwork has an almost negligible influence on the hydration heat temperature characteristics of the precast concrete segmental box girder.

(2) The thickness of plastic formwork has a limited effect on the absolute increase in the peak temperature of precast concrete segmental box girders. It significantly increases the thermal inertia of the precast concrete segmental box girder, thereby affecting the cooling rate and stress development after the formwork removal. When using formwork with good thermal insulation for precast concrete segmental box girders, the dual effects of formwork thickness on peak temperature and cooling rate after formwork removal should be considered comprehensively. As wind speed increases, the temperature peak of the precast concrete segmental box girder shifts to approximately 0.5 days earlier. The maximum temperature of the precast concrete segmental box girder is positively correlated with the casting temperature; higher casting temperatures lead to earlier temperature peaks and greater thermal stresses, exhibiting maximum temperature and thermal stress sensitivities of 12.7% and 21.7%, respectively. The layout of prestressing ducts significantly affects peak temperatures and thermal stresses at the beam ends of precast concrete segmental box girders. Segments without ducts experience higher maximum stresses than those with ducts. In contrast, the presence or absence of ducts has little effect on the temperature or thermal stress of the midspan segment of precast concrete segmental box girders.

(3) The distribution characteristics of the early-age temperature field of the precast concrete segmental box girder are similar under the different refined curing control measures. Due to the large volume at the junction between the top slab and the web of the precast concrete segmental box girder, heat dissipation is slow, leading to heat accumulation, a larger internal–external temperature difference, and consequently greater early-age thermal stresses. Therefore, appropriate curing measures should be implemented to reduce the risk of cracking in precast concrete segments.

(4) Sprinkler curing in summer effectively reduces the internal–external temperature difference of the concrete and lowers early-age thermal stress. In winter, steam curing enhances the early strength of concrete, delays the peak temperature, maximum temperature difference, and peak thermal stress, thereby avoiding the critical cracking period and reducing the risk of thermal cracking. These results verify the rationality and feasibility of the refined curing control strategy. Moreover, in both winter and summer, the peak thermal stress occurs approximately 1 day after the maximum temperature, with a corresponding temperature difference, indicating a lag effect.

## Data Availability

The authors declare that the data supporting the findings of this study are available in the article.
